# Assessment of conformity of actual thoraco-lumbar pedicle screw dimensions to manufacturers’ specifications

**DOI:** 10.1177/00368504211035035

**Published:** 2021-08-16

**Authors:** Marian Banas, Nirjhar Hore, Michael Buchfelder, Sebastian Brandner

**Affiliations:** 1 Department of Neurosurgery, University Hospital Erlangen, Erlangen, Germany; 2 Department of Neurosurgery, Clinic Hohe Warte, Bayreuth, Germany

**Keywords:** Pedicle screws, spinal instrumentation, biomechanics

## Abstract

Although correct selection of pedicle screw dimensions is indispensable to achieving optimum results, manufacturer-specified or intended dimensions may differ from actual dimensions. Here we analyzed the reliability of specifications made by various manufacturers by comparing them to the actual lengths and diameters of pedicle screws in a standardized experimental setup. We analyzed the actual length and diameter of pedicle screws of five different manufacturers. Four different screw lengths and for each length two different diameters were measured. Measurements were performed with the pedicle screws attached to a rod, with the length determined from the bottom of the tulip to the tip of the screw and the diameters determined at the proximal and distal threads. Differences in length of > 1 mm were found between the manufacturers’ specifications and our actual measurements in 24 different pedicle screws. The highest deviation of the measured length from the manufacturers’ specification was 3.2 mm. The difference in length between the shortest and longest screw with identical specifications was 3.4 mm. The highest deviation of the measured proximal thread diameters and the manufacturer’s specifications was 0.5 mm. The diameter of the distal thread depends on the shape of the pedicle screw and hence varies between manufacturers in conical screws. We found clear differences in the length of pedicle screws with identical manufacturer specifications. Since differences between the actual dimensions and the dimensions indicated by the manufacturer may vary, this needs to be taken into account during the planning of spine instrumentation.

## Introduction

Transpedicular instrumentation is a frequent procedure for dorsal stabilization of the thoraco-lumbar spine. Pullout strength of inserted pedicle screws depends on the design characteristics of the screws, the bone structure and quality, the accuracy of screw placement, and insertion depth.^1–3^ Fixation in the pedicle contributes most to the strength of the fixation.^4^ Accurate selection of the screw diameter is essential to achieve optimal insertion into the pedicle and avoid fracturing of the cortical bone around the pedicle.^5^

In relation to insertion depth, in most cases avoidance of anterior cortical breach is crucial due to risk of neurovascular injury. In some cases, however, intentional bicortical fixation might be justified in the interest of increased stability.^1^

Pedicle screws consist of two elements, the tulip and the shaft. Morphology of the shaft is characterized by its shape, which can be cylindrical or conical, and by its dimensions. The dimensions of the shaft are specified by length and diameter, the latter can be given as the outer diameter of the thread and the inner diameter of the shaft without the thread. To achieve optimal screw fixation, exact knowledge of screw dimensions is essential. Dimensions of pedicle screws are usually indicated as *diameter* × *length* by the manufacturers. However, due to missing standards to adhere to, the actual dimensions might vary across manufacturers due to different measurement paradigms. The aim of this study was to analyze the lengths and diameters of thoraco-lumbar pedicle screws of different manufacturers in a standardized manner and compare our results with the manufacturers’ specifications.

## Materials and methods

No ethical approval was required for this in vitro biomechanical study.

We analyzed the actual length and diameter of pedicle screws for thoraco-lumbar instrumentation from five different manufacturers (Neo Medical—Neo Pedicle Screw System™, HumanTech—Venus^®^, Aesculap—S4^®^ Spinal System, DePuySynthes—Expedium^®^, K2M—Everest^®^) in an experimental setup. Four different lengths (35, 40, 45, and 50 mm) and for each length two different diameters (5 and 6 mm or 5.5 and 6.5 mm or 6 and 7 mm) as indicated by the manufacturers were measured. For each type, two identical screws were analyzed.

Measurements were performed with a vernier caliper with the pedicle screws attached to a rod. Screw length was determined from the bottom of the tulip to the tip of the screw in order to ensure a uniform method of measurement of pedicle screws by different manufacturers. The pedicle screw diameters were determined at the proximal and distal threads. The results were then compared to the manufacturers’ specifications.

## Results

**Figure 1.**
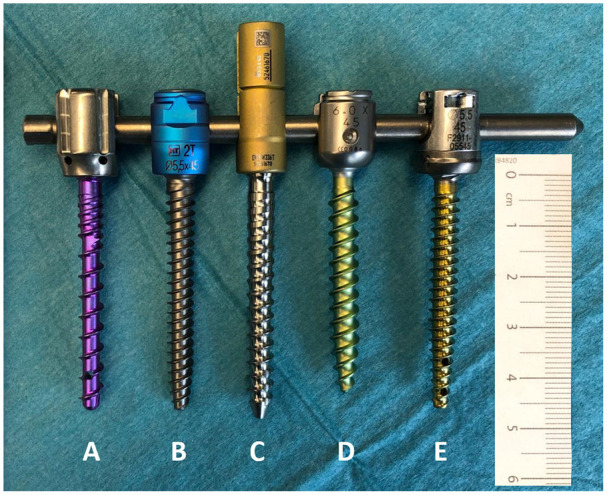
Pedicle screws of the dimension 5.0 mm, 5.5 mm, or 6 mm × 45 mm attached with their tulips to a rod, where the highest discrepancies in length are noted. The variation in length between identically labeled pedicle screws is illustrated in the differing ends of screw tips: (a) Neo Medical—Neo Pedicle Screw System™, (b) HumanTech—Venus^®^, (c) Aesculap—S4^®^ Spinal System, (d) DePuySynthes—Expedium^®^, and (e) K2M—Everest^®^.

A detailed overview of the results is depicted in Table 1. No differences were detected between two identical screws of any given manufacturer. Differences in length of >1 mm between the manufacturers’ specifications and our measurements were found in 24 different pedicle screws. The highest deviation of the measured length from the manufacturers’ specification was 3.2 mm in screws with the specification 35 mm × 5.5 mm and 45 mm × 5.5 mm (Figure 1). The difference in length between the shortest and longest screw with a supposedly identical specification of length by the respective manufacturers was 3.4 mm (shortest: DePuySynthes—Expedium^®^35 mm × 7 mm; longest Aesculap—S4^®^ Spinal System 35 mm × 5.5 mm).

**Table 1.** Consolidated overview of complete data gathered.

Length manufacturer (mm)	Length measured(mm)	Ø Proximalthread (mm)	Ø Distalthread (mm)	Lengthmanufacturer(mm)	Lengthmeasured(mm)	Ø Proximalthread (mm)	Ø Distalthread (mm)
Neo Medical—Neo Pedicle Screw System™							
Ø5.0				Ø6.0			
35	35.5	5.0	5.0	35	35.6	6.0	6.0
40	40.5	5.0	5.0	40	40.5	6.0	6.0
45	45.5	5.0	5.0	45	45.6	6.0	6.0
50	50.6	5.0	5.0	50	50.6	6.0	5.9
HumanTech—Venus^®^							
Ø5.5				Ø6.5			
35	37.0	5.8	4.5	35	37.0	6.9	5.0
40	42.0	5.8	4.4	40	42.0	6.9	5.0
45	47.0	5.9	4.5	45	47.0	7.0	5.0
50	51.9	5.9	4.6	50	52.0	7.0	4.8
Aesculap—S4^®^ Spinal System							
Ø5.5				Ø6.5			
35	38.2	5.4	5.4	35	38.0	6.4	6.3
40	43.0	5.4	5.3	40	43.0	6.4	6.3
45	48.2	5.4	5.2	45	48.0	6.4	6.4
50	53.1	5.4	5.4	50	52.8	6.4	6.4
DePuySynthes—Expedium^®^							
Ø6.0				Ø7.0			
35	35.0	5.7	4.3	35	34.8	6.8	5.7
40	40.0	5.8	4.6	40	40.0	6.8	5.8
45	44.9	5.8	4.8	45	45.1	6.8	5.9
50	50.0	5.7	4.5	50	49.9	6.8	5.6
K2M—Everest^®^							
Ø5.5				Ø6.5			
35	36.4	5.3	3.9	35	36.5	6.4	4.5
40	41.5	5.3	4.0	40	41.6	6.3	4.4
45	46.6	5.4	4.0	45	46.5	6.4	4.5
50	51.7	5.3	4.1	50	51.7	6.2	4.7

The labeled diameter of pedicle screws conformed to the diameter of the proximal thread in all screws, irrespective of whether they were of conical or cylindrical shape. The highest deviation of the measured proximal thread diameter and the manufacturer’s specification was 0.5 mm. The diameter of the distal thread depends on the shape of the pedicle screw. The proximal and distal diameters of the cylindrical pedicle screws were identical across all manufacturers. In conical-shaped screws, the dimensions of the distal thread diameter vary from manufacturer to manufacturer.

## Discussion

Characteristics of pedicle screws have a crucial impact on the biomechanics and strength of pedicle screw-based spinal instrumentation. The main features of pedicle screws are characterized by their length and their diameter, parameters which are clearly labeled on the screws by the manufacturers.^1,2^ However, the actual dimensions of pedicle screws with the same specifications might vary between different manufacturers due to different measurement modalities. In this study, we, therefore, analyzed the lengths and diameters of pedicle screws of various manufacturers in a standardized manner. We found notable discrepancies in length between pedicle screws with the same dimension specification of up to 3.4 mm.

Approximately 60% of the pullout strength of vertebral screw fixation depends on the fixation in the pedicle.^4^ The pullout strength in turn is proportionate to the diameter of the screw utilized and the intrinsic bone density.^6^ However, to maintain fixation strengths, the fracturing of the cortical bone around the pedicle must be avoided. This must especially be taken into account in osteoporotic patients with a thinner cortical bone, with screws with a too-large diameter posing a high risk of fracturing it and thus impairing stability.^7–9^ Accurate fitting of screws in the pedicle is thus crucial for achieving optimal results.^10^

While exact planning of screw insertion depth is crucial for obtaining good strengths, this is of critical importance when a bicortical fixation is planned, as engagement of the anterior cortex has been shown to significantly increase the pullout strengths and improve bone fixation especially in osteoporotic patients.^11,12^ However, when penetrating the anterior cortex exact knowledge of the pedicle screw dimensions is crucial to avoid violation of anterior structures which could lead to vascular injuries.^13,14^

Therefore it is imperative for a surgeon to be exactly aware of the diameter of the utilized screws in the given pedicle segment. As opposed to clear deviations of screw lengths among different manufacturers, we did not find notable deviations of the diameters of the proximal threads in our measurements. The diameter of the proximal thread generally conformed to the labeled diameter on the screws, representing the largest individual screw shaft diameter in conical-shaped screws.

Our present study focused on differences in lengths and diameters of pedicle screws with the same labeling between different manufacturers. There are also other screw design elements significantly influencing biomechanics and stability of pedicel screw-based spinal instrumentation, such as the thread design or the shaft shape, which were not analyzed in this study due to manifold variations making a clear systematization impossible.^15,16^ Nevertheless, surgeons must take possible advantages or shortcomings into account. Our current data shows that due to a lack of uniform standards regulating the labeling of screw dimensions across various manufacturers, surgeons may not be able to rely on specified and labeled dimensions alone and would need to take relevant deviations into consideration.

## Conclusion

Since we found dimensions of pedicle screws to exhibit relevant differences between different manufacturers despite bearing the same specifications, this might become of critical value when surgeons change instrumentation systems. Being accustomed to the specific dimensions of pedicle screws of one manufacturer, switching to another manufacturer might lead to unexpected results when actual screw dimensions may differ from the formerly used screws.

At present, there are no uniform standards for defining dimensions in the manufacture of pedicle screws. Since these dimensions of different manufacturers do vary, we recommend the introduction of standards that would be obligatory for all manufacturers to adhere to.
